# *Mycobacterium avium* subsp. *paratuberculosis* and associated risk factors for inflammatory bowel disease in Iranian patients

**DOI:** 10.1186/s13099-016-0151-z

**Published:** 2017-01-03

**Authors:** Samin Zamani, Mohammad Reza Zali, Hamid Asadzadeh Aghdaei, Leonardo Antonio Sechi, Magdalena Niegowska, Elisa Caggiu, Rouhollah Keshavarz, Nader Mosavari, Mohammad Mehdi Feizabadi

**Affiliations:** 1 Department of Microbiology, School of Medicine, Tehran University of Medical Sciences, Tehran, Iran; 2 Gastroenterology and Liver Diseases Research Center, Shahid Beheshti University of Medical Sciences, Tehran, Iran; 3 Basic and Molecular Epidemiology of Gastrointestinal Disorders Research Center, Research Institute for Gastroenterology and Liver Diseases, Shahid Beheshti University of Medical Sciences, Tehran, Iran; 4 Department of Biomedical Sciences, University of Sassari, Viale San Pietro 43b, 07100 Sassari, Italy; 5 PPD Tuberculin Department, Razi Vaccine & Serum Research Institute, Karaj, Iran; 6 Reference Laboratory for Bovine Tuberculosis, Razi Vaccine and Serum Research Institute, Agricultural Research Education and Extension Organization (AREEO), Tehran, Iran; 7 Thoracic Diseases Research Center, Tehran University of Medical Sciences, Tehran, Iran

**Keywords:** *Mycobacterium avium* subsp. *paratuberculosis*, Inflammatory bowel disease, Crohn’s disease, ELISA, IS900 nested PCR, Iran

## Abstract

**Background:**

Inflammatory bowel disease (IBD) is described as a relapsing condition with high morbidity and uncertain complex pathogenesis. The association of *Mycobacterium avium* ssp. *paratuberculosis* (MAP) with Crohn’s disease (CD) in human has been debated for decades, however there is no confirmed data to verify such relations in Iran. The aim of this study was to investigate risk factors and a possible role of MAP in Iranian patients with CD.

**Methods:**

Anti-MAP antibodies were detected in serum of IBD patients and subjects without IBD (nIBD) through ELISA; MAP DNA and viable MAP cells were identified in patients’ biopsies through nested PCR and direct culture methods, respectively. Principal component analysis (PCA) was used to investigate the risk factors in relation to IBD and MAP infection.

**Results:**

Positivity for IS900 PCR was detected in 64% (n = 18) of CD, 33% (n = 10) of ulcerative colitis (UC) and 9.7% (n = 6) of nIBD samples. Live MAP cells were isolated from biopsies of 2 CD patients only. Among 28 patients with CD, 46% (n = 13) and 39% (n = 11) were positive for antibodies against MAP3865c_133–141_ and MAP3865c_125–133_ peptides, respectively, whereas much lower seroreactivity was detected in UC subjects accounting for 3% (n = 1) for MAP3865c_133–141_ and 16.7% (n = 5) for MAP3865c_125–133_. A high immune reactivity to MAP epitopes among CD patients was positively correlated with consumption of fast food meals and IBD familiarity. For both CD and UC, breastfeeding period and consumption of fruit/vegetables presented negative correlation with the presence of anti-MAP antibodies.

**Conclusions:**

This study provided evidences that high prevalence of MAP DNA and anti-MAP antibodies in CD patients is significantly associated with the development of CD. Despite the role of several factors contributing to IBD, the presence of MAP DNA and anti-MAP antibodies in Iranian CD patients highlights a possible transmission of MAP from animal-derived products to humans.

## Background

Crohn’s disease (CD) and ulcerative colitis (UC) are the main types of inflammatory bowel disease (IBD) described as a relapsing condition with high morbidity and uncertain pathogenesis. In genetically susceptible persons cell-mediated immune responses to gastrointestinal bacteria seem to be involved in the etiology of these complex disorders [1–3]. The populations of North America and Europe are more affected by IBD than Asians [2], however the incidence of IBD has been rising in either Asia or developing countries [4]. Likewise, IBD has been diagnosed more recurrently in different parts of Iran in the last decades [5, 6]. In line with the high incidence of IBD in American and European countries, most information regarding epidemiology, symptoms, risk factors and etiology of IBD are described based on the local populations [7, 8]. Although the etiology of IBD is largely complex and unknown, it is thought to be caused by a combination of genetic and environmental factors that affect the immune responses. Among the environmental factors, *Mycobacterium avium* subsp*. paratuberculosis* (MAP)—a causative agent of Johne’s disease (JD) in domestic and wild ruminants, seems to be important in the pathogenesis of IBD, despite conflicting reports on its possible role in the disease. Some studies showed the involvement of MAP in CD in genetically susceptible individuals [9–11], whereas other reports could not confirm such findings [12–14].

There are limited data describing these complex disorders in countries like Iran, while existence of MAP in industrial and traditional cattle farms, its isolation from feces, milk of dairy herds and from lymph nodes of goats along with undesirable consequences in ruminant livestock have been reported in various parts of the country [15–17]. During subclinical phase of JD MAP is shed through feces and milk, and can spread within the herd [18]. The ability of MAP to survive on vegetables fertilized with contaminated manure, in water, raw meat, milk and dairy products is widely described. Therefore, infected animals are considered as an important source for transmission to humans [19–22]. Even though the association between MAP and Crohn’s disease highlighting its putative triggering role has been intensely debated, there is no confirmation to verify similar relation in Iran [23]. Likewise, the knowledge regarding risk factors for IBD and rates of MAP infection in the country have not been cleared yet. Given the wide diffusion of the mycobacterium in domestic breeding and dairy industry in Iran, the need to study epidemiologic features and the impact of MAP on IBD is fundamental.

In this study, the presence of MAP in IBD patients and nIBD controls was investigated through nested-PCR, ELISA and culture methods. Additionally, correlation of MAP infection with IBD risk factors such as age, sex, clinical symptoms (diarrhea, constipation, bloody stool, low Hb and lose weight), breastfeeding and dietary habits including milk and meat consumption, was determined.

## Methods

### Patients and tissue specimens

In the present study, 120 specimens from IBD and non-IBD (nIBD) patients were collected. The study population was classified as follows: 28 patients affected by CD (14 males and 14 females; mean age 39.1 years, range 18–64), 30 patients affected by UC (12 males and 18 females; mean age 36.9 years, range 16–75) and 62 nIBD individuals (29 males and 33 females; mean age 50.35 ears, range 18–76). The protocols were approved by Ethics Committee of Tehran University of Medical Sciences and the informed consent was obtained in all cases. The diagnosis of CD was based on conventional clinical, endoscopic, and histologic criteria and disease activity was assessed with Crohn’s Disease Activity Index (CDAI) [24, 25]. Biopsy samples were obtained from the affected colon, terminal ileum and rectum of patients upon colonoscopy. UC patients presented typical features such as active colitis with ulceration and inflammation of the mucosa with special distribution. The selected patients were newly diagnosed for IBD and have not undergone antibiotic treatment for at least 3 months. nIBD controls were selected among individuals subjected to screening surgery due to other nIBD-associated conditions like abdominal pain or an alteration in bowel habit. No colonoscopic or histological abnormalities of the ileum or colon were observed in this group. Any kind of clinical and/or histopathological diagnosis of IBD was excluded and the biopsies were obtained in the same way as did for patients with IBD/CD or UC. No history of antibiotic therapy during 3 months prior to sampling is recorded. Additionally, 2 ml of blood samples collected from all subjects were transferred into clot activator tubes and centrifuged for 10 min at 400×*g* (2000 rpm) in order to separate the serum for further ELISA tests. The sera were preserved at −20 °C until use.

### Bacterial culture of gut specimens

All biopsy samples were resuspended in 15 ml of freshly prepared sterile 0.75% (w/v) hexadecylpyridinium chloride (HPC; Merck, Darmstadt, Germany) and kept at room temperature (25 °C) for 18 h. After decontamination samples were centrifuged at 3000×*g* for 20 min and the sediments were resuspended in 5 ml PBS. Equal volume of each decontaminated sample was inoculated into Herrold’s egg yolk medium (HEY) containing mycobactin J (MJ) and incubated at 37 °C for up to 6 months.

### DNA extraction

DNA was extracted directly from biopsies using RTP^®^ Mycobacteria Kit (Invitek, Berlin, Germany) and stored at −20 °C for nested-PCR assays.

### IS900 specific nested PCR and sequencing

MAP target gene IS900 was amplified using nested PCR primers [L/AV] L (L1: 5′-CTT TCT TGA AGG GTG TTC GG-3′ and L2: 5′-ACG TGA CCT CGC CTC CAT-3′) and AV (AV1: 5′-ATG TGG TTG CTG TGT TGG ATG G-3′ and AV2: 5′-CCG CCG CAA TCA ACT CCA G-3′) according to protocols described by other authors [26]. Then, specificity of the amplicons was checked by sequencing.

### ELISA

MAP3865c_125–133_ (MIAVALAGL) and MAP3865c_133–141_ (LAANFVVAL) peptides were synthesized at >90% purity (GL Biochem). The peptides were resuspended in 10 mM of dimethyl sulfoxide (DMSO) and kept in single-use aliquots at −70 °C. Indirect enzyme-linked immunosorbent assay (ELISA) was used to detect anti-MAP3865c_133–141_ specific antibodies (Abs). 10 µg/ml of MAP3865c peptides diluted in 0.05 M carbonate–bicarbonate buffer (Sigma) used for coating of 96-well Nunc immunoplates. Phosphate-buffered saline (PBS) containing 0.05% Tween-20 (PBS-T) was used as washing buffer, modified by adding 5% non-fat dried milk (Sigma) for blocking of nonspecific binding sites. All steps were performed as previously reported [27]. VersaTunable MAX microplate reader was applied for determination of optical density (OD) at the wavelength of 405 nm.

### Risk factor variables

IBD risk factors analyzed in correlation with MAP infection in the present study population [28] were selected based on demographic data (age, sex, geographical provenience), clinical symptoms (diarrhea, constipation, bloody stool, low Hb and lose weight), ongoing therapy, disease familiarity, period of breastfeeding, smoking and dietary habits (consumption of raw and pasteurized milk, fast food meals, green tea, fruit and vegetables).

### Statistical analysis

The cut-off values for sample positivity were calculated based on the receiver operating characteristics (ROC) curve with specificity established at 95%. In case of CD patients, cut-off points equaled 0.45 U/ml for MAP3865c_133–141_ and 0.73 U/ml for MAP3865c_125–133_. For UC subjects, the threshold values were established at 0.50 and 0.73 U/ml for MAP3865c_133–141_ and MAP3865c_125–133_ epitopes, respectively. The same nIBD individuals were used as a reference for the analysis of both disease groups. Statistical significance of the data was determined through the student’s *t* test (95% CI) employing GraphPad Prism software (version 6.02, La Jolla, USA). Variables correlated with the positivity for MAP peptides were identified through the principal component analysis using XLSTAT (version 2015.1, Addinsoft, France). The cut-off for variable loadings describing the degree of correlation between variables and principal components was arbitrarily set at ≥0.45 as previously described [29], with higher values considered major contributors.

## Results

**Fig. 1:**
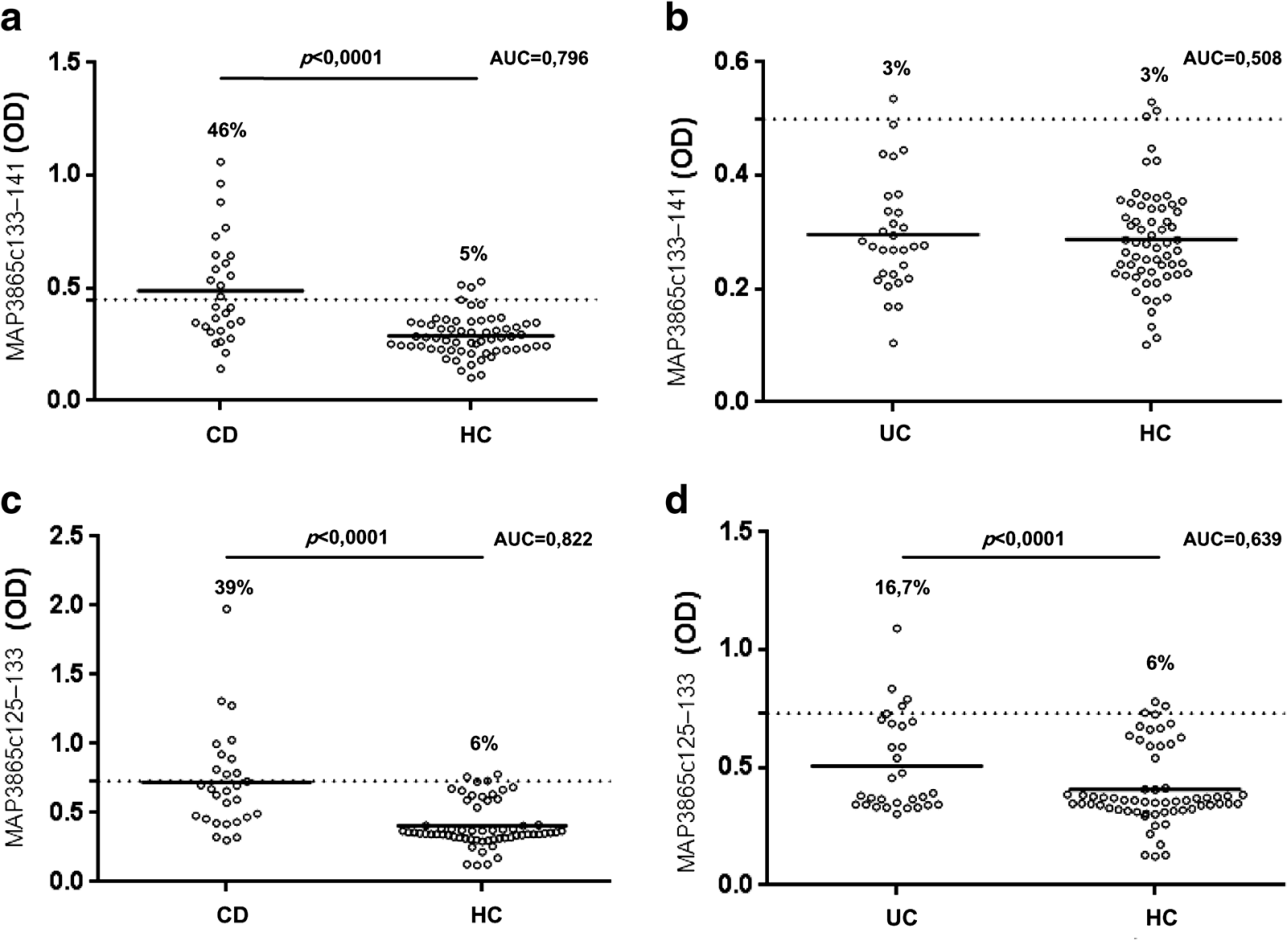
Prevalence of Abs against MAP epitopes in CD, UC and nIBD subjects. Distribution of Abs values based on the statistical analyses was performed separately for MAP3865c_133–141_ (**a**, **b**) and MAP3865c_125–133_ (**c**, **d**) with respect to disease. The *dotted lines* indicate the cut-off for positivity relative to each assay established through ROC analysis. The percentage of anti-MAP reactive subjects, statistically significant *p* values (CI 95%) and AUC are reported on top of each distribution. *Horizontal bars* specific for CD, UC and nIBD groups represent means

Among 28 CD patients, 46% (n = 13) were positive for MAP3865c_133–141_ and 39% (n = 11) for MAP3865c_125–133_ peptides (*p* < 0.0001 in both cases), whereas much lower seroreactivity was detected among UC subjects accounting for 3% (n = 1) for MAP3865c_133–141_ and 16.7% (n = 5) for MAP3865c_125–133_ (*p* < 0.69 and *p* < 0.0067, respectively). nIBD displayed anti-MAP positivity of 5% (n = 3) for MAP3865c_133–141_ and 6% (n = 4) for MAP3865c_125–133_ epitopes when the analysis was statistically significant (Fig. 1).

**Fig. 2:**
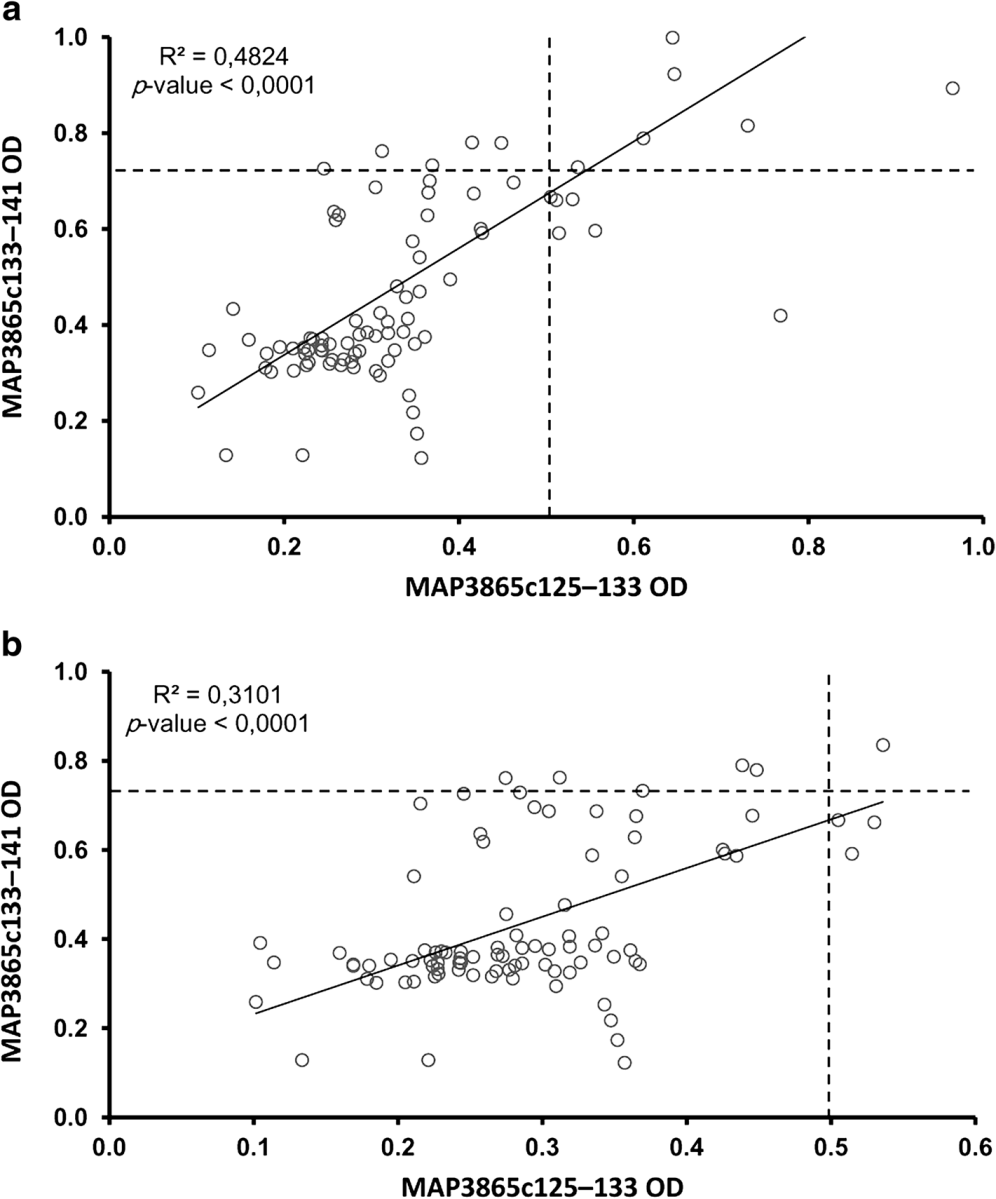
Correlation between Abs recognizing MAP3865c_133–141_ and MAP3865c_125–133_ peptides in CD, UC and nIBD subjects. Distributions corresponding to CD (**a**) and UC (**b**) were analyzed with the same nIBD group. *Circles* correspond to patients or nIBD positive to anti-MAP Ab. Thresholds for positivity calculated by ROC analysis and specific to each assay are indicated by the *dotted lines*. R2 and p values relative to each correlation are reposted in the *top-left* corners

The peptides highly correlated in CD patients (R^2^ = 0.48; Fig. 2) characterized by positivity to both antigens in 60% of individuals bearing anti-MAP Abs. This correlation was weaker for UC subjects (R^2^ = 0.31). Upon sex-related analysis, Abs reactivity against any of the MAP peptides among males and females was equal in CD patients and nIBD but showed 2:3 ratio in UC subjects.

MAP was cultured in Herrold’s egg medium plus mycobactin J from biopsies of two CD patients, both of them were positive in ELISA and PCR tests.

MAP specific IS900 gene was detected by nested PCR in 64% of CD (n = 18), 33% of UC (n = 10) and 9.7% of nIBD (n = 6) samples; the IS900 positivity was not reflected by the presence of anti-MAP Abs in 11 cases among patients only, however 6 of them showed high Abs titers (Table 1). All the biopsy specimens with positive MAP DNA detection were taken from the terminal ileum.

**Table 1:** Prevalence of anti-MAP Abs and MAP DNA among CD, UC and nIBD patients

Diagnosis	anti-MAP1 Abs+	anti-MAP1 Abs−	anti-MAP2 Abs+	anti-MAP2 Abs−	IS900+	IS900−
CD	13 (46%)	15 (54%)	11 (39%)	17 (61%)	18 (64%)	10 (36%)
UC	1 (3%)	29 (97%)	5 (16.7%)	25 (83.3%)	10 (33%)	20 (67%)
nIBD	3 (5%)	59 (95%)	4 (6%)	58 (94%)	7 (9.7%)	55 (90.3%)

Numbers with the relative percentages of subjects positive (Abs+) and negative (Abs−) to MAP3865c_133–141_ (MAP1) and MAP3865c_125–133_ (MAP2) peptides. Analogous data referred to detection of MAP-specific IS900 gene is provided for each group

Multivariate analysis revealed several variables among demographic data, clinical history and nutritional habits correlated with MAP epitopes. Following the Keiser–Guttman criterion, ten principal components with eigenvectors ≥1.0 were identified for CD patients and accounted for 70.3% of the total variation, however variable loadings exceeding the established cut-off were not found in the last principal component (Table 2). For principal component 1 described by 19% of the variation, 12 variables showed loadings higher compared to the established threshold, translated into a strong relationship between immune reactivity to the MAP peptides, presence of MAP DNA, IBD history within family, Asacol treatment, consumption of fast food meals and clinical symptoms such as bloody stool, low Hb and weight loss; an inverse association was observed among variables related to age, consumption of fruit and vegetables and duration of breastfeeding period. Three values reached the cut-off point for principal component 2 accounting for 8.5% of the total variation and included familiarity of cancer, consumption and pasteurization of milk. Principal components from 3 to 9 were described each by only one variable.

**Table 2:** Loadings of variables most related to each principal component representative for CD patients and nIBD

Variables	Principal components								
	PC1	PC2	PC3	PC4	PC5	PC6	PC7	PC8	PC9
Total variation (%)	18.89	8.57	7.9	7.26	5.7	5.15	4.65	4.56	4.08
MAP3865c_133–141_	0.65	−0.15	−0.32	−0.28	0.15	0.17	−0.17	0.04	0.01
MAP3865c_125–133_	0.74	−0.21	−0.28	−0.28	0.01	0.21	−0.19	−0.05	−0.02
MAP DNA	0.74	0.10	−0.21	−0.10	0.12	0.03	−0.20	0.15	−0.03
Age	−0.66	0.28	−0.32	−0.03	−0.29	0.06	−0.2	−0.11	0.15
Gender	−0.15	−0.09	−0.5	0.63	0.24	−0.06	−0.27	0.03	0.06
City	0.06	−0.23	−0.18	−0.25	−0.25	−0.31	−0.14	0.48	−0.31
IBD history	0.49	0.28	0.25	0.38	−0.39	0.04	−0.01	−0.02	0.03
Cancer history	0.02	0.46	−0.36	−0.11	0.18	0.01	0.35	0.1	−0.04
Diabetes history	0.21	0.38	−0.46	−0.05	−0.19	−0.24	0.27	0.2	0.01
Asacol treatment	0.48	0.13	0.32	0.12	−0.08	0.52	−0.12	0.19	0.07
Diarrhea	0.24	0.29	−0.09	−0.24	0.49	−0.15	0.21	−0.14	0.09
Constipation	−0.07	−0.25	−0.22	0.32	−0.06	0.19	0.46	−0.38	0.01
Bloody stool	0.47	−0.37	−0.04	0.13	−0.2	−0.21	0.21	−0.18	−0.1
Low Hb	0.54	−0.17	−0.27	0.2	−0.08	0.14	0.16	−0.33	−0.26
Weight loss	0.52	0.33	0.01	0.21	0.32	−0.26	0.07	0.17	−0.04
Smoke	0.22	0.4	−0.13	−0.28	−0.02	−0.01	0.08	−0.19	0.52
Milk consumption	0.15	−0.45	0.07	0.15	−0.23	0.29	0.11	0.16	0.28
Pasteurized milk	−0.15	0.52	0.15	0.3	0.19	0.36	−0.29	−0.26	−0.02
Fruit/vegetables	−0.57	−0.06	−0.13	−0.15	0.35	−0.08	−0.12	−0.15	−0.05
Breastfeeding duration	−0.56	0.03	−0.28	−0.36	0.02	0.15	0.03	−0.21	−0.15
Fast food	0.48	0.06	0.25	0.11	0.46	0.00	−0.04	0.11	0.05

Nine principal components with major contribution of each variable have been included. Italic values denote loadings ≥0.45. The table includes only variables exceeding the established threshold, relative to demographics, clinical history and nutritional habits of the selected participants. Percentage of the total variation is given for each principal component

For UC patients, nine principal components were identified with the cumulative variation of 67.6% and loadings higher than ≥1.0 observed in the first eight components. Principal component 1 was explained by 18% of the total variation and 9 variables including reactivity against MAP3865c_125–133_ only, IBD history, Mesalamine treatment, clinical symptoms characterized by diarrhea, bloody stool and weight loss. Age, consumption of fruit and vegetables as well as breastfeeding period also presented a strong relationship but in the opposite way. Correlation between the two MAP peptides was characteristic for principal component 2 described by 9.5% of the total variation and, as expected, presented an inverse relationship with patients’ gender. Of note, consumption of green tea was negatively associated with anti-MAP Abs positivity, clinical symptoms and history of IBD and diabetes which may be due to its antibacterial effect.

**Fig. 3:**
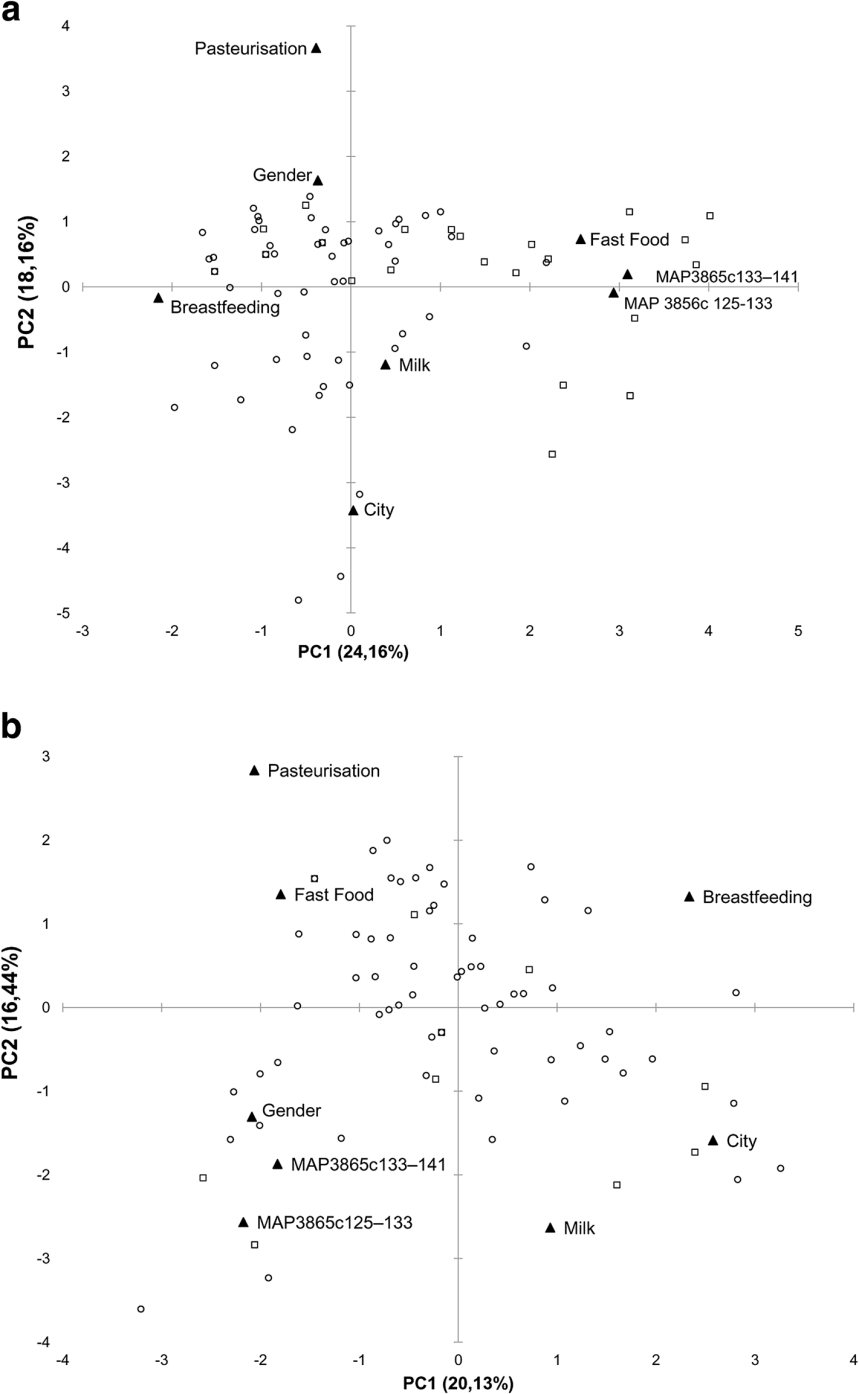
Principal component analysis of variables with potential influence on MAP transmission and positivity to MAP3865c_133–141_/MAP3865c_125–133_ peptides. Bi-plots show correlation between the analyzed epitopes and variables relative to personal dietary history, geographical provenience and gender in CD patients (**a**), UC subjects (**b**) and nIBD used for both analyses. Samples positive to anti-MAP Abs are represented by *squares* whereas those negative by *circles*; all variables are described by *labels* and their position is indicated by *triangles*. The distribution illustrates only PC1 and PC2 correlations

Based on several reports associating MAP to diet and its possible transmission with contaminated food, we restricted our analysis to variables describing patients’ nutritional habits, geographical provenience and duration of breastfeeding. Figure 3 illustrates bi-plot distributions of variables and Abs positivity to MAP epitopes in both patients groups and nIBD. A strong relationship was observed between MAP antigens and frequency of fast food meals consumption, mirrored by a higher reactivity to the peptides. This relationship was explained by 42% of the total variation for CD patients and 35% for UC subjects for whom the fast food variable described principle component 3. In both cases, milk pasteurization was not negatively correlated with positivity to anti-MAP Abs pointing at the ability of the mycobacterium to survive this process. Duration of breastfeeding showed an inverse correlation indicating its protective role against MAP infection. The prevalence of women among UC patients reactive to MAP epitopes was explained by principal component 1.

## Discussion

CD and UC mainly involve the gastrointestinal tract. Individuals with susceptible gene loci exposed to certain environmental factors undergo the activation of immune responses to a variety of species composing the gut microbiota that results in the development of CD or UC [30]. Similar to other developing countries, the rate of UC and CD is increasing in Iran [4]. This study described a high prevalence of MAP DNA in biopsies of CD patients. Among CD individuals, the positivity for IS900 PCR—a specific repeated insertion sequence in MAP genome, was characterized by high statistical significance suggesting a possible role of MAP in triggering CD, whereas lower IS900 PCR detection rates were observed in UC patients. Moreover, most of the samples in the control group were negative for MAP DNA. Products of the positive samples were sequenced and further confirmed the presence of MAP. Our results point at the isolation of MAP from livestock and dairy products reported in Iran and suggest a possible exposure to the microorganism. Infected animals with subclinical symptoms of JD, in particular dairy cattle, shed MAP from feces and may increase the risk of its transmission to humans [16, 17]. The absence of MAP DNA in some CD patients may be explained by the fact that different bacterial populations are responsible for CD in distinct intestinal regions. The positive results obtained by PCR did not match with those of ELISA in 11 patients suggesting that sensitivity of ELISA test was lower than PCR, as described in a previous study [31]. Additionally, history of IBD treatment may lower anti-MAP Abs titers; to note is the fact that nIBD individuals showed a complete coincidence of serological response and molecular results. Several reports demonstrated that MAP can be more commonly detected from CD than UC or healthy populations [32–34]. Conversely, some studies have not found an association between MAP detection and IBD [35, 36].

Following 6 months of incubation in culture media, only two samples with positive ELISA and PCR results simultaneously showed colonies of MAP; poor culture outcomes may be due to the fastidious nature of the mycobacterium that is hardly detected by conventional microbiological techniques [37].

In CD patients, the positivity rates for MAP3865c peptides were higher compared to UC subjects and nIBD group. As the specificity of the bacterial peptides applied in our ELISA method achieves a 100% for MAP, we can certainly declare that ELISA-positive subjects were exposed to MAP and not to other environmental mycobacteria. In some previous studies, the association between anti-MAP peptides and autoimmune diseases like CD, type 1 diabetes, multiple sclerosis (MS) and Hashimoto’s thyroiditis have been reported [38–41].

Detection of increased seroreactivity against MAP in few control samples may be caused by environmental exposure to the extracellular form of MAP; however, production of preservative Abs against MAP in these subjects potentially prevents the progression to CD [22].

In this study, results of nested PCR and ELISA showed a significant association of positivity to antibodies against MAP and the presence of MAP DNA in a group of Iranian CD patients. A similar association has been described in several studies including different populations. Culture was positive from biopsies of two CD patients only. Nevertheless, MAP DNA was also detected in UC and nIBD controls with a lower prevalence in comparison to CD subjects. These data permit to hypothesize that MAP may not be the only etiological factor, but probably a predisposing element in interplay with other determinants.

In some clinical trials, anti-mycobacterial therapy including clarithromycin, rifabutin and/or clofazimine had positive effects for the treatment of CD and MS patients. If the role of MAP is confirmed, we can recommend prescription of anti-mycobacterial therapy that may result more effective in practice for Iranian IBD patients under treatment of CD [42].

A high immune reactivity to MAP epitopes among CD patients was positively correlated with the consumption of fast food meals, weight loss, milk pasteurization and IBD familiarity. For both CD and UC, factors such as age, breastfeeding period and consumption of fruit/vegetables presented negative correlation with the presence of anti-MAP antibodies. The results of our study showed that breastfeeding influences MAP colonization and IBD onset in adulthood. According to some authors, breastfeeding has a protective effect on IBD development, especially when continued for at least 12 months [43, 44]. The beneficial role of breastfeeding most likely relates to changes in gut microbial community in stool of breast-fed infants compared to those fed with milk formula [43]. Also, according to previous studies, dysbiosis of the fecal microbiota can be linked with MAP infection [45]. Such changes in gut microbial community are correlated as well with the increasing rate of IBD; particularly, frequent consumption of fast food meals, fatty acids and reduced intake of fruit and vegetables have been associated with the disease development [43–46]. Moreover, polyphenols contained in fruit, and at high concentrations in green tea, can help to increase the levels of antioxidants and attenuate severity of colitis in the analogous way to sulfasalazine, reducing the rate of IBD in Iranians [43, 47]. The result of this study proposes that pasteurized milk would need to be considered as a weak transmission vector confirming the previous report [48] and is in line with other reports. Data regarding the intake of dairy products were not available for the enrolled patients; consumption of processed cheese has been significantly associated with CD and its evaluation in Iranian cohort would be worthwhile [49].

## Conclusions

Despite the role of multiple factors in progression to IBD, detection of anti-MAP antibodies and MAP DNA together with two cases of isolation of viable MAP cells from Iranian CD patients highlights the importance of possible sources of exposure to the mycobacterium and routes of its transmission from animal products to humans. Yet, MAP may be a predisposing factor according to a high significance of the obtained data; additional information about the genetic background of patients and role of other bacteria and environmental factors are needed for a complete picture. If MAP role in CD is approved, it can be a warning for health care organizations to improve hygiene controlling systems in livestock farms and dairy industries towards reduction of MAP transmission to humans. Also, for these complex disorders, the optimal application of antibiotics indicated for a defined patient group will depend on a clear determination of microbial agents involved in the pathogenesis. The present study suggests that anti-mycobacterial therapy should be more effective in practice. Enrollment of more numerous patient groups from various geographical regions of Iran may further elucidate the role of MAP in IBD.

## Abbreviations

IBD: inflammatory bowel disease
MAP: *Mycobacterium avium* ssp. *paratuberculosis*
CD: Crohn’s disease
UC: ulcerative colitis
PCA: principal component analysis
ELISA: enzyme-linked immunosorbent assay
Abs: antibodies
PBS: phosphate-buffered saline
DMSO: dimethyl sulfoxide
ROC: operating characteristics
JD: Johne’s disease

## References

[1] Podolsky DK (2002). Inflammatory bowel disease. N Engl J Med.

[2] Selby W (2000). Pathogenesis and therapeutic aspects of Crohn’s disease. Vet Microbiol.

[3] Xavier RJ, Podolsky DK (2007). Unravelling the pathogenesis of inflammatory bowel disease. Nature.

[4] Gismera CS, Aladren BS (2008). Inflammatory bowel diseases: a disease(s) of modern times? Is incidence still increasing?. World J Gastroenterol.

[5] Aghazadeh R, Zali MR, Bahari A, Amin K, Ghahghaie F, Firouzi F (2005). Inflammatory bowel disease in Iran: a review of 457 cases. J Gastroenterol Hepatol.

[6] Daryani NE, Bashashati M, Aram S, Hashtroudi AA, Shakiba M, Sayyah A, Nayer-Habibi A (2006). Pattern of relapses in Iranian patients with ulcerative colitis. A prospective study. J Gastrointestin Liver Dis.

[7] Wang YF, Zhang H, Ouyang Q (2007). Clinical manifestations of inflammatory bowel disease: East and West differences. J Dig Dis.

[8] Yang SK, Loftus EV, Sandborn WJ (2001). Epidemiology of inflammatory bowel disease in Asia. Inflamm Bowel Dis.

[9] Scanu AM, Bull TJ, Cannas S, Sanderson JD, Sechi LA, Dettori G (2007). *Mycobacterium avium* subspecies *paratuberculosis* infection in cases of irritable bowel syndrome and comparison with Crohn’s disease and Johne’s disease: common neural and immune pathogenicities. J Clin Microbiol.

[10] Taylor H (2009). *Mycobacterium avium* subspecies *paratuberculosis*, Crohn’s disease and the Doomsday scenario. Gut Pathog.

[11] Sechi LA, Scanu AM, Molicotti P, Cannas S, Mura M, Dettori G (2005). Detection and isolation of *Mycobacterium avium* subspecies *paratuberculosis* from intestinal mucosal biopsies of patients with and without Crohn’s disease in Sardinia. Am J Gastroenterol.

[12] Rowbotham DS, Mapstone NP, Trejdosiewicz LK, Howdle PD, Quirke P (1995). *Mycobacterium paratuberculosis* DNA not detected in Crohn’s disease tissue by fluorescent polymerase chain reaction. Gut.

[13] Frank TS, Cook SM (1996). Analysis of paraffin sections of Crohn’s disease for *Mycobacterium paratuberculosis* using polymerase chain reaction. Mod Pathol.

[14] Clarkston WK, Presti ME, Petersen PF, Zachary PE, Fan WX, Leonardi CL (1998). Role of *Mycobacterium paratuberculosis* in Crohn’s disease. Dis Colon Rectum.

[15] Ansari-Lari M, Haghkhah M, Bahramy A, Baheran AM (2009). Risk factors for *Mycobacterium avium* subspecies *paratuberculosis* in Fars province (Southern Iran) dairy herds. Trop Anim Health Prod.

[16] Pourjafar M, Badiei K. Presence and trace element analysis in cattle affected by paratuberculosis in Yasouj, Iran. In: 8th international colloquium on paratuberculosis, Spain. 2005.

[17] Khodakaram Tafti A, Rashidi K (2000). The pathology of goat paratuberculosis: gross and histopathological lesions in the intestines and mesenteric lymph nodes. J Vet Med.

[18] Bhide M, Chakurkar E, Tkacikova L, Barbuddhe S, Novak M, Mikula I (2006). IS900-PCR-based detection and characterization of *Mycobacterium avium* subsp. *paratuberculosis* from buffy coat of cattle and sheep. Vet Microbiol.

[19] Szewzyk U, Szewzyk R, Manz W, Schleifer KH (2000). Microbiological safety of drinking water. Annu Rev Microbiol.

[20] Le Dantec C, Duguet JP, Montiel A, Dumoutier N, Dubrou S, Vincent V (2002). Occurrence of mycobacteria in water treatment lines and in water distribution systems. Appl Environ Microbiol.

[21] Taylor RH, Falkinham JO, Norton CD, LeChevallier MW (2000). Chlorine, chloramine, chlorine dioxide, and ozone susceptibility of *Mycobacterium avium*. Appl Environ Microbiol.

[22] Hermon-Taylor J, El-Zaatari FAK. The *Mycobacterium avium* subspecies *paratuberculosis* problem and its relation to the causation of Crohn disease. In: Bartram J, Cotruvo J, Dufour A, Rees G, Pedley S, editors. Pathogenic Mycobacteria in water: a guide to public health consequences, monitoring and management. London: IWA Publishing; 2004. p. 74–94.

[23] Grant IR, Rowe MT, Dundee L, Hitchings E (2001). *Mycobacterium avium* ssp. *paratuberculosis*: its incidence, heat resistance and detection in milk and dairy products. Int J Dairy Technol.

[24] Dignass A, Eliakim R, Magro F, Maaser C, Chowers Y, Geboes K (2012). Second European evidence-based consensus on the diagnosis and management of ulcerative colitis part 1: definitions and diagnosis. J Crohns Colitis.

[25] Dignass A, Van Assche G, Lindsay JO, Lémann M, Söderholm J, Colombel JF (2010). The second European evidence-based consensus on the diagnosis and management of Crohn’s disease: current management. J Crohns Colitis.

[26] Bull TJ, McMinn EJ, Sidi-Boumedine K, Skull A, Durkin D, Neild P (2003). Detection and verification of *Mycobacterium avium* subsp. *paratuberculosis* in fresh ileocolonic mucosal biopsy specimens from individuals with and without Crohn’s disease. J Clin Microbiol.

[27] Masala S, Paccagnini D, Cossu D, Brezar V, Pacifico A, Ahmed N (2011). Antibodies recognizing *Mycobacterium avium paratuberculosis* epitopes cross-react with the beta-cell antigen ZnT8 in Sardinian type 1 diabetic patients. PLoS ONE.

[28] Jakobsen C, Paerregaard A, Munkholm P, Wewer V (2013). Environmental factors and risk of developing paediatric inflammatory bowel disease—a population based study 2007–2009. J Crohns Colitis..

[29] Hsu FC, Kritchevsky SB, Liu Y, Kanaya A, Newman AB, Perry SE (2009). Association between inflammatory components and physical function in the health, aging, and body composition study: a principal component analysis approach. J Gerontol Ser A Biol Sci Med Sci.

[30] Baumgart DC, Sandborn WJ (2012). Crohn’s disease. Lancet.

[31] Collins MT, Lisby G, Moser C, Chicks D, Christensen S, Reichelderfer M (2000). Results of multiple diagnostic tests for *Mycobacterium avium* subsp. *paratuberculosis* in patients with inflammatory bowel disease and in controls. J Clin Microbiol.

[32] Behr MA, Kapur V (2008). The evidence for *Mycobacterium paratuberculosis* in Crohn’s disease. Curr Opin Gastroenterol.

[33] Abubakar I, Myhill D, Aliyu SH, Hunter PR (2008). Detection of *Mycobacterium avium* subspecies *paratuberculosis* from patients with Crohn’s disease using nucleic acid-based techniques: a systematic review and meta-analysis. Inflamm Bowel Dis.

[34] Feller M, Huwiler K, Stephan R, Altpeter E, Shang A, Furrer H (2007). *Mycobacterium avium* subspecies *paratuberculosis* and Crohn’s disease: a systematic review and meta-analysis. Lancet Infect Dis.

[35] Naser SA, Ghobrial G, Romero C, Valentine JF (2004). Culture of *Mycobacterium avium* subspecies *paratuberculosis* from the blood of patients with Crohn’s disease. Lancet.

[36] Ricanek P, Lothe SM, Szpinda I, Jorde AT, Brackmann S, Perminow G (2010). Paucity of mycobacteria in mucosal bowel biopsies from adults and children with early inflammatory bowel disease. J Crohns Colitis.

[37] Green EP, Tizard ML, Moss MT, Thompson J, Winterbourne DJ, McFadden JJ (1989). Sequence and characteristics of IS900, an insertion element identified in a human Crohn’s disease isolate of *Mycobacterium paratuberculosis*. Nucleic Acids Res.

[38] Shariati SH, Alaei A, Keshavarz R, Mosavari N, Rabbani A, Niegowska M (2016). Detection of *Mycobacterium avium* subsp. *paratuberculosis* in Iranian patients with type 1 diabetes mellitus by PCR and ELISA. J Infect Dev Ctries.

[39] Cossu D, Masala S, Frau J, Cocco E, Marrosu MG, Sechi LA (2013). Anti *Mycobacterium avium* subsp. *paratuberculosis* heat shock protein 70 antibodies in the sera of Sardinian patients with multiple sclerosis. J Neurol Sci.

[40] Elsaghier A, Prantera C, Moreno C, Ivanyi J (1992). Antibodies to *Mycobacterium paratuberculosis*-specific protein antigens in Crohn’s disease. Clin Exp Immunol.

[41] Niegowska M, Paccagnini D, Burrai C, Palermo M, Sechi LA (2015). Antibodies against proinsulin and homologous MAP epitopes are detectable in Hashimoto’s thyroiditis Sardinian patients, an additional link of association. PLoS ONE.

[42] Sechi LA, Dow CT (2015). *Mycobacterium avium* ss. *paratuberculosis* Zoonosis—the hundred year war-beyond Crohn’s disease. Front Immunol..

[43] Ng SC, Tang W, Leong RW, Chen M, Ko Y, Studd C (2015). Environmental risk factors in inflammatory bowel disease: a population-based case–control study in Asia-Pacific. Gut.

[44] Hansen TS, Jess T, Vind I, Elkjaer M, Nielsen MF, Gamborg M (2011). Environmental factors in inflammatory bowel disease: a case–control study based on a Danish inception cohort. J Crohns Colitis.

[45] Fecteau ME, Pitta DW, Vecchiarelli B, Indugu N, Kumar S, Gallagher SC (2016). Dysbiosis of the fecal microbiota in cattle infected with *Mycobacterium avium* subsp*. paratuberculosis*. PLoS ONE.

[46] Hou JK, Abraham B, El-Serag H (2011). Dietary intake and risk of developing inflammatory bowel disease: a systematic review of the literature. Am J Gastroenterol.

[47] Oz HS, Chen T, de Villiers WJ (2013). Green tea polyphenols and sulfasalazine have parallel anti inflammatory properties in colitis models. Front Immunol.

[48] Anzabi Y, Hanifian S (2012). Detection of *Mycobacterium avium* subspecies *paratuberculosis* in pasteurized milk by IS900 PCR and culture method. Afr J Microbiol Res.

[49] Waddell L, Rajić A, Stärk K, McEwen SA (2016). *Mycobacterium avium* ssp. *paratuberculosis* detection in animals, food, water and other sources or vehicles of human exposure: a scoping review of the existing evidence. Prev Vet Med.

